# Impaired mitochondrial morphology and respiratory dysfunction in human induced pluripotent stem cells with mitochondrial tRNA mutations (m.3243A>G and m.14739G>A)

**DOI:** 10.1186/s13023-026-04201-z

**Published:** 2026-01-29

**Authors:** Fibi Meshrkey, Kelly M. Scheulin, Bibhuti Saikia, Joshua Stabach, Raj R. Rao, Franklin D. West, Shilpa Iyer

**Affiliations:** 1 Department of Biological Sciences, Fulbright College of Arts and Sciences, University of Arkansas, Fayetteville, AR USA; 2 Cell and Molecular Biology Program, University of Arkansas, Fayetteville, AR USA; 3 Department of Histology and Cell Biology, Faculty of Medicine, Alexandria University, Alexandria, Egypt; 4 Regenerative Bioscience Center, University of Georgia, Athens, GA USA; 5 Department of Animal and Dairy Science, University of Georgia, Athens, GA USA; 6 Biomedical and Health Sciences Institute, Neuroscience Program, University of Georgia, Athens, GA USA; 7 Department of Biomedical Engineering, College of Engineering, University of Arkansas, Fayetteville, AR USA; 8 Department of Biological Sciences, J. William Fulbright College of Arts and Sciences, Science and Engineering 601, Fayetteville, AR 72701 USA

**Keywords:** Mitochondrial disease, tRNA mutation, hiPSC, Pluripotent stem cell, Reprogramming, Bioenergetics, Respiration, Mitochondrial dynamics, Mitochondrial morphology

## Abstract

**Background:**

Mitochondrial DNA (mtDNA) mutations contribute to respiratory dysfunction and cause mitochondrial diseases. The pathologies of these multisystemic inherited diseases are poorly understood. Mutations in the mitochondrial tRNA gene are one of the most frequent mtDNA mutations and are associated with various clinical symptoms such as diabetes mellitus, hearing loss, cardiomyopathy, exercise intolerance, and found in patients with mitochondrial disorders. Human induced pluripotent stem cells (hiPSCs), generated by reprogramming patient-specific somatic cells are recognized as useful tools for disease modeling and serve to better understand the multisystemic pathologies associated with mitochondrial tRNA mutations.

**Methods:**

hiPSCs were generated from control BJ and two fibroblast lines with mitochondrial tRNA mutations using non-modified reprogramming and immune evasion mRNAs and microRNAs. Expression of hiPSC-associated intracellular and cell surface markers were identified by immunofluorescence and flow cytometry. Sanger sequencing was used to detect the mutation in all hiPSCs. Mitochondrial network morphology analysis was conducted to detect and quantify mitochondrial structures. The mitochondrial respiration ability, glycolytic function, and composite bioenergetics health index (BHI) were measured by the Seahorse Bioscience XFe96 extracellular flux analyzer.

**Results:**

Reprogrammed hiPSCs expressed pluripotent stem cell markers including transcription factors POU5F1, NANOG, and SOX2, and cell surface markers SSEA4, TRA-1-60, and TRA-1-81 at the protein level. Sanger sequencing analysis confirmed the presence of mutations in both hiPSCs. Cytogenetic analyses confirmed the presence of normal karyotypes in both hiPSCs. Mitochondrial morphological analysis indicates the presence of hyperfused mitochondria in diseased hiPSC lines. The composite BHI values, a measure of mitochondrial dysfunction that was based on comprehensive bioenergetics analysis of OXPHOS and glycolysis, demonstrated that the mitochondrial functional defects were severe in both the hiPSC lines exhibiting tRNA mutations.

**Conclusion:**

Overall, the hiPSCs exhibited variable mitochondrial morphology and respiratory dysfunction that have the potential to alter hiPSC differentiation potential, cell fate, and tissue development. These results indicate the potential significance of using hiPSCs and their derivatives to assess mitochondrial morphology and key bioenergetics parameters as a means to better understand early developmental defects in mitochondrial disorders in children.

**Supplementary Information:**

The online version contains supplementary material available at 10.1186/s13023-026-04201-z.

## Background

Mutations in mitochondrial DNA (mtDNA) are associated with a wide variety of maternally inherited genetic disorders that aggravate mitochondrial dysfunction and contribute to human diseases with complex pathologies such as developmental delays, brain damage, cardiomyopathy, lactic acidosis, autism, and infertility [1–4]. Since the initial discovery of the first mtDNA mutation in the context of mitochondrial diseases [5–7]. More than 400 pathogenic mutations have been characterized [1, 3, 8], with numerous studies demonstrating the potential for these point mutations to not only alter specific electron transport chain (ETC) proteins, but also the structure and entire multiprotein assembly, thus leading to bioenergetic and cellular dysfunction [4, 9–13].

Among the many mutations, a common mitochondrial disorder caused by a point mutation in mtDNA is MELAS (mitochondrial encephalomyopathy, lactic acidosis, and stroke-like episodes) syndrome. In most cases, MELAS syndrome is caused by a transition from adenine to guanine in position 3243 (m.3243A>G) in the mt-tRNA^Leu.UUR^ (*MT-TL1*) gene [14]. The specific 3243A>G mutation affects mt-tRNA structure stabilization, methylation, aminoacylation, and triplet recognition [15]. The mutation causes a defect of UUG-rich gene translation that results in a specific complex I deficiency that is characteristic of MELAS syndrome [16]. Although MELAS syndrome is primarily associated with neurological symptoms, it affects several organs with clinical manifestations including stroke-like episodes, dementia, epilepsy, lactic acidemia, myopathy, recurrent headaches, hearing impairment, diabetes, and short stature [17]. Another novel and possibly de novo mutation worthy of investigation is the 14739G > A mutation in the mt-tRNA^Glu^ (*MT-TE*) gene. The detected m.14739G > A mutation is located in the central position of the aminoacyl acceptor stem of the tRNA^Glu^ and introduces a third non-standard U-G base pairing. This additional nonstandard base pairing probably destabilizes the acceptor stem and is supported by the absence of comparable constellations in tRNA^Glu^ of other mammalian species [18]. In a study conducted with a patient who exhibited loss of appetite, weakness, and exercise intolerance, the pathogenic m.14739G > A mutation was identified to various degrees in different tissues, depending on tissue-specific cytochrome c oxidase activity [19].

Although mtDNA encodes 22 tRNAs necessary for the translational process within mitochondria, the genetic and biochemical consequences of many pathogenic tRNA mutations are currently unknown in early development. There is growing interest in studying the impact of these tRNA mutations as these result in combined respiratory chain deficiency of complexes I, III, as well as cytochrome c oxidase (COX), and affect the ATP synthase to a certain degree. Therefore, the creation of patient-specific human induced pluripotent stem cells (hiPSCs) containing defined mtDNA mutations related to mitochondrial disorders [20–26] is worthy of further study as it will provide key insights into the role of the mitochondrial genome and accompanying bioenergetic dysfunction in early embryonic development and disease progression. Previous studies demonstrated that isogenic hiPSCs reprogrammed from MELAS syndrome (m.3243A>G) patient fibroblasts using viral vectors exhibited variable mutant mtDNA levels, which influence cell fate and mitochondrial function [23, 27–30]. We have recently demonstrated the ability to use non-viral approaches to reprogram patient fibroblasts containing mtDNA mutations to generate hiPSCs that continue to exhibit the mutation, while maintaining differentiation potential [20, 22]. Despite these advances, many questions remain, including the morphological and functional deficits caused by tRNA mutations in mtDNA.

In this study, we have used a combination of non-modified reprogramming and immune evasion mRNAs and microRNAs to generate hiPSCs from two patient fibroblasts containing (a) the classic MELAS syndrome m.3243A>G mutation affecting the *MT*-*TL1* gene of the tRNA leucine and (b) a novel m.14739G > A mutation affecting the *MT-TE* gene of the tRNA glutamic acid [19]. We fully characterized the generated hiPSCs using (a) immunocytochemical and flow cytometry approaches; (b) cytogenetic analyses to demonstrate normal karyotype; (c) Sanger sequencing to detect the mutation burden; (d) mitochondrial network morphology analysis to detect and quantify mitochondrial structures; and (e) thoroughly assessed the mitochondrial respiration and glycolytic function to study the biochemical consequences of mtDNA-tRNA mutations during early development.

## Materials and methods

### Ethics statement

The current study was conducted with patient fibroblasts provided by the Medical University of Salzburg (SBG), Austria. Informed consent was obtained to use these samples for research in an anonymized way. In accordance with federal regulations regarding the protection of human research subjects (32 CFR 219.101(b) [4]), the University of Arkansas Office of Research Compliance determined that the project was exempt from Institutional Review Board (IRB) oversight and human research subjects’ protection regulations.

### Cell culture

Cultures of control BJ (ATCC^®^ CRL-2522™) fibroblasts (ATCC, Manassas, VA, USA) and two patient-derived diseased fibroblasts (SBG6 (m.3243A>G), SBG7 (m.14739G > A)) were obtained from the Medical University of Salzburg, Austria. These cells were maintained in fibroblast expansion medium that consisted of minimal essential medium (MEM) (Thermo Fisher Scientific, Waltham, MA, USA), 10% fetal bovine serum (FBS) (GE Healthcare - HyClone™, Chicago, IL, USA), and 2mM L-glutamine. Fibroblasts were enzymatically passaged in 0.05% Trypsin-EDTA (Thermo Fisher Scientific, Waltham, MA, USA).

Once reprogrammed, hiPSCs were maintained in NutriStem hPSC xeno-free (XF) medium (Biological Industries, Cromwell, CT, USA) with Stemolecule Y27632 Dihydrochloride Hydrate (Reprocell, Beltsville, MD, USA) on a highly purified and refined laminin-511 E8 fragment matrix, iMatrix-511 (Reprocell, Beltsville, MD, USA) on a 24-hour feeding schedule. hiPSCs were enzymatically passaged once reaching 70–80% confluency at a split ratio of 1:3 using StemPro^®^ Accutase^®^ (Thermo Fisher Scientific, Waltham, MA, USA). Both fibroblast and hiPSC cultures were maintained without the use of antibiotics, handled in Biosafety Type II sterile hoods regularly cleaned with UV irradiation and 70% ethanol, and grown in 37 °C incubators at 5% CO2 and 95% humidity.

### Somatic cell reprogramming to hiPSCs using a combination of non-modified reprogramming and immune evasion RNAs

**Fig. 1:**
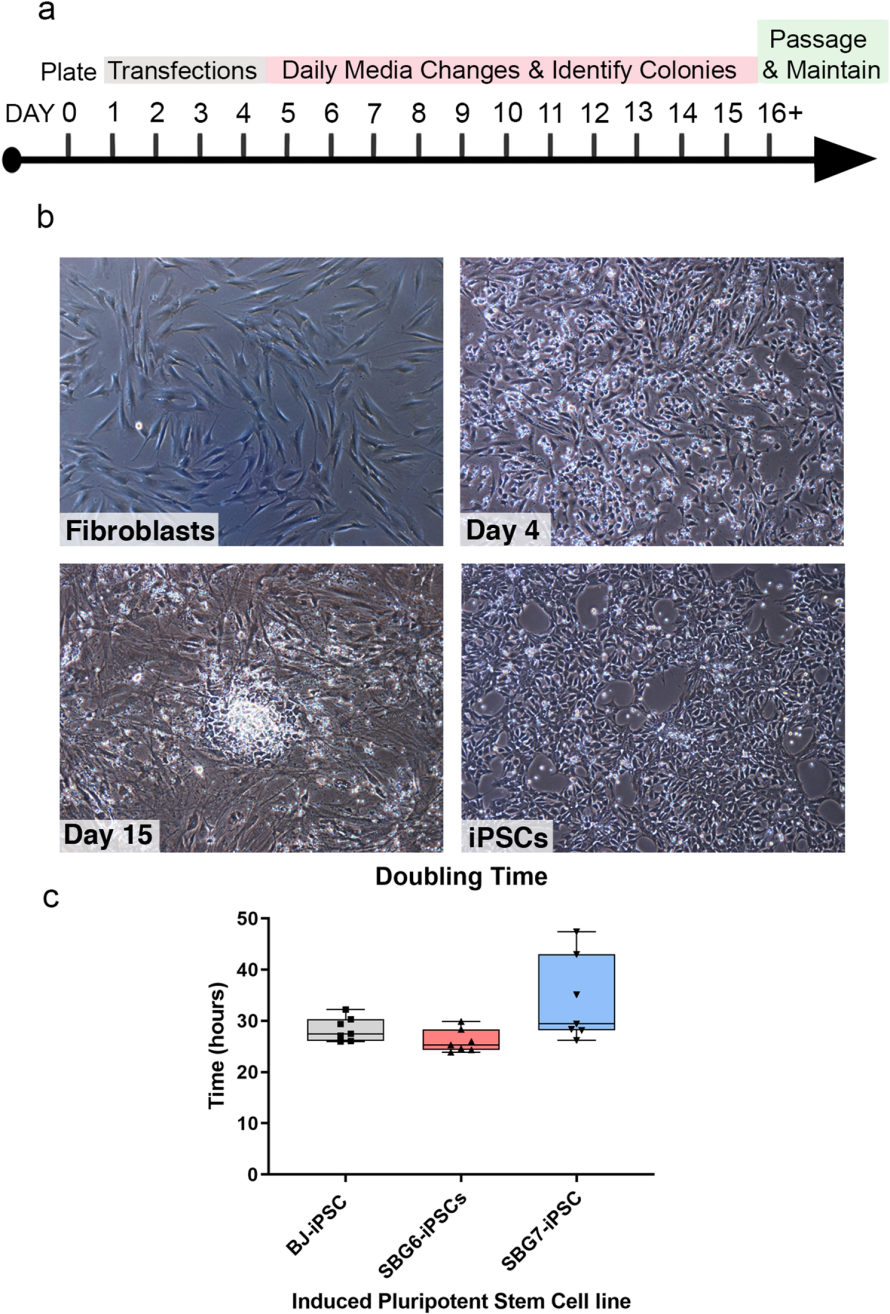
Human patient dermal fibroblasts reprogrammed into hiPSCs using mRNA–microRNA approach. (**a**) Reprogramming schematic for mRNA and microRNA transfections. (**b**) Bright field images (10 x) of human disease fibroblasts undergoing reprogramming. Day 0 fibroblasts displayed flat and elongated morphology typical of fibroblasts. Day 4 transfected cells show morphological changes, including a rounded morphology and apoptosis, which are consistent with the reprogramming process. Multipolar spindle-shaped fibroblast cells transitioning to a more compact cobblestone appearance at day 10, with early stem cell colonies arising by day 11–13. Day 15 cultures show colony formation, a key iPSC characteristic when cells are in contact with fibroblasts, with well-circumscribed borders. Isolated stem cells maintain characteristic well-circumscribed morphology with minimal spontaneous differentiations through 20 + passages. (**c**) Doubling time analyses indicate that all hiPSCs exhibit similar proliferation times representative of actively dividing cells

Putative hiPSCs were generated using the StemRNA 3rd Generation Reprogramming Kit for Adult and Neonatal Human Fibroblasts (Reprocell, Beltsville, MD, USA). Briefly, 1 × 10^5^ fibroblasts were plated in 35 mm dishes in fibroblast expansion medium on iMatrix extracellular substrate at day 0. On day 1, culture medium was switched to NutriStem and a reprogramming cocktail was added to the culture for 4 days of overnight transfections (Fig. 1). The reprogramming cocktail consisted of non-modified microRNAs for the reprogramming factors (POU5F1 (aka OCT4), SOX2, Klf4, cMyc, Nanog, Lin28), and immune evasion factors (E3, K3, B18) [31]. It is useful to note that mRNA reprogramming results in the generation of genetically stable hiPSCs, while avoiding the risks of genomic integration. However, non-modified synthetic mRNAs are immunogenic and activate cellular defense mechanisms, which can lead to cell death and inhibit mRNA translation upon repetitive transfection. To overcome RNA-related toxicity, we have incorporated immune evasion RNAs as part of our reprogramming cocktail [31]. Daily medium changes were performed until colonies were large enough to be isolated. At day 13–18, putative hiPSCs were identified and enzymatically passaged with StemPro^®^ Accutase^®^ (Thermo Fisher Scientific, Waltham, MA, USA) and maintained in culture for over 4–9 passages until apoptosis decreased. The newly created hiPSCs were identified as (SBG6- (m3243A > G) hiPSC, SBG7- (m.14739G > A)-hiPSC). Doubling time assay was performed using hiPSCs between passages 11 and 12 by manual counts (*n* = 3) at 12, 24, 36 and 48 h after plating. Population doubling time was determined using exponential regression curve fitting (https://www.doubling-time.com/compute_more.php).

### Immunocytochemical analysis

For immunocytochemical detection of the pluripotency markers POU5F1, SOX2, SSEA4, TRA-1-60, and TRA-1-81, cells were cultured in NutriStem medium on iMatrix-coated 4 well Permanox^®^ slides (Nunc Lab-Tek^®^ Chamber Slide™ System; Thermo Fisher Scientific, Waltham, MA, USA). Cells were fixed with 4% paraformaldehyde (PFA) solution. For intracellular epitope antibody staining, fixed cells were permeabilized with blocking solution containing 0.3% Triton X-100, 1% polyvinylpyrrolidone, and 3% chicken serum in PBS with Ca^2+^ and Mg^2+^. Intracellular staining utilized a PBS with Ca^2+^ and Mg^2+^ wash buffer containing 0.05% Tween 20. For extracellular epitopes, fixed cells were blocked in PBS with Ca^2+^ and Mg^2+^ containing 6% chicken serum and washed with PBS with Ca^2+^ and Mg^2+^. Primary antibodies used for immunocytochemical hiPSC pluripotency characterization were POU5F1/OCT4 (Santa Cruz, Dallas, TX, USA sc-5279 (mouse) or sc-9081 (rabbit); 1:200), SOX2 (R&D Systems, Minneapolis, MN, USA; MAB2018; 1:200), SSEA4 (Invitrogen, Waltham, MA, USA, MA1-021; 1:50), TRA-1-60 (Stemgent, Beltsville, MD, USA, 09–0068; 1:50), TRA-1-81 (Stemgent, Beltsville, MD, USA, 09–0069; 1:50). Primary antibodies were detected by fluorophore-labeled secondary antibodies Alexa Fluor 488 (Invitrogen, Waltham, MA, USA; 1:1000) and Alexa Fluor 594 (Invitrogen, Waltham, MA, USA; 1:1000). Nuclei were stained and slides were mounted with Prolong Gold (Invitrogen, Waltham, MA, USA) with 4’6-diamidino-2-phenylindole (DAPI) (1:1000). Slides were imaged on a Zeiss LSM 880 confocal microscope.

### Flow cytometry analysis

For flow cytometry analysis of multiple pluripotency markers, cells were fixed in 4% PFA and blocked in PBS without Ca^2+^ and Mg^2+^ in 6% chicken serum. For intracellular marker detection, cells were permeabilized three times by adding 0.05% Tween 20 in the block solution. Cells were blocked for 45 min at room temperature. Primary antibodies against POU5F1/OCT4 (Santa Cruz, Dallas, TX, USA; sc-5279; 1:200), SOX2 (R&D Systems, Minneapolis, MN, USA; MAB2018; 1:200), Nanog (Invitrogen, Waltham, MA, USA; PA1-097; 1:200), SSEA4 (Invitrogen, Waltham, MA, USA; MA1-021; 1:50), TRA-1-60 (Stemgent, Beltsville, MD, USA; 09–0068; 1:50), and TRA-1-81 (Stemgent, Beltsville, MD, USA; 09–0069; 1:50) were used for flow cytometry. Primary antibodies were detected using fluorescently conjugated secondary antibody Alexa Fluor 488 (Invitrogen, Waltham, MA, USA; 1:1000) and Alexa Fluor 594 (Invitrogen, Waltham, MA, USA; 1:1000). Cells were analyzed using the Quanteon analyzer (Agilent, Santa Clara, CA, USA) and FlowJo cytometry analysis software (Tree Star, Ashland, OR, USA).

### Cytogenetic analysis

Upon reaching a 50%-60% confluence, cell cultures were treated with KaryoMAX colcemid (Thermo Fisher Scientific, Waltham, MA, USA) at a final concentration of 0.1 µg/mL for 40 min. Cells were subsequently enzymatically detached using StemPro^®^ Accutase^®^ (Thermo Fisher Scientific, Waltham, MA, USA) with Accutase inactivation achieved through resuspension in Nutristem medium. Cells were then spun for 10 min at 500 g and resuspended in a heated hypotonic solution of 0.075 M KCl for 30 min, followed by fixation with 3:1 methanol to glacial acetic acid. Fixed cells were stored at 4 °C for at least 24 h before resuspending in 0.5 mL of 3:1 methanol to glacial acetic acid fixative. Cells were dropped onto slides and dried over a humidity zone created using a heat block with a layer of damp paper towel. Slides were then aged 24 h in a 55 °C oven. Giemsa banding patterns were produced using pancreatin and Wright’s stain, with FBS being used to inactivate the pancreatin. Images were taken using a 100x objective mounted on an Evos FL inverted microscope. Karyotypes were produced from images using the SmartType software platform.

### DNA isolation and purification

Frozen cell pellets containing ~ 2 × 10^6^ cells were thawed and processed. The QIAamp DNA mini kit (Qiagen, Valencia, CA, USA) manufacturer protocol was followed to extract total DNA, which resulted in an elution of 100 µL of distilled water (dH2O) and total DNA from all cells. The 100 µL solutions containing the genomic DNA were further treated with 1.0 µL of RNaseA for 1 h at 37 °C to avoid RNA contamination. The gDNA was quantified using DeNovix UV/Vis Spectrophotometer (DeNovix Inc. Wilmington, DE, USA). A blank of 1.0µL of dH2O was used to establish a zero, and 1.0 µL of each sample was used to determine the concentration.

### PCR and sanger sequencing analysis

Primers for use in PCR were generated using the human mtDNA sequence provided by mitomap.org/MITOMAP, and IDT’s Primer Quest tool (IDT, Coralville, Iowa, USA) and Primer 3 (https://bioinfo.ut.ee/primer3/). A standard PCR was carried out using the Takara Taq PCR Amplification Kit (Clontech Mountain View, CA, USA). The targeted gene region was amplified using the primer pairs shown in Supplementary Table 1. PCR was performed in a total volume of 50 µl, containing 25 µl, master mix (Promega, Madison, WI, USA), 0.2 µmol of each primer, and 100–400 ng of genomic gDNA.

Thermal cycling conditions were 5 min at 95 °C, followed by 36 cycles [30 s at 95 °C, 30 s at the annealing temperature of the primers (52–65 °C) and 30 s at 72 °C] and a final extension for 5 min at 72 °C and a hold at 4 °C for infinity. After PCR reactions, the PCR products were run on 2% agarose gel and DNA band was excised. Further, the gel purification was carried out using QIAEX II Gel extraction Kit (Qiagen, Germantown, MD, USA). The DNA was quantified using DENOVIX, spectrophotometer. Finally, the purified PCR products were shipped for Sanger sequencing to UAMS sequencing core. Samples were sequenced at the UAMS Sequencing Core Facility using a 3500 Genetic Analyzer (Applied Biosystems, Foster City, CA, USA). The genotyping and fragment sizing were done on a 3130XL (Applied Biosystems, Foster City, CA, USA). The files generated from the sequencing were observed using CodonCode Aligner (CodonCode Corporation, Centerville, MA, USA).

### Mitochondrial oxygen consumption detection and glycolysis function test, and bioenergetics health index (BHI)

Although metabolic shifting is essential for successful reprogramming, mitochondria still play an important role in regulating the fate of hiPSCs [32–36]. In this study, we evaluated the metabolic state in the parental fibroblasts and the generated hiPSCs to further understand the influence of mitochondrial genome perturbations on cellular bioenergetics. Changes in oxygen consumption were measured in real time using XFe96 extracellular flux analyzer. Seahorse XF96 Cell Mito Stress Test Kit and glycolytic rate assay kit (Seahorse Biosciences, USA) were used as per the manufacturer’s instructions. Corning^®^ Cell-Tak™ Cell and Tissue Adhesive (Corning, NY, USA) was used to immobilize the cells (following the manufacturer’s procedure) for non-adherent cells. Before use in XFe96, hiPSCs were detached using Accutase and seeded into the coated plate with a previously optimized number of 25,000 cells per well. All hiPSCs were seeded in eight replicate wells per plate, with the experiment repeated no less than 3–5 times.

The cells were supplemented with 180 µl Mito-stress complete Seahorse medium, after which the cells were incubated in a non-CO_2_ incubator at 37 °C for one hour. Respiration was measured using the classic mitochondrial inhibitors, specific for complex I and III subunits, such as Rotenone and Antimycin A (0.5 µM final concentrations each). Maximum respiration was measured by the addition of an uncoupler (Carbonyl cyanide-p-trifluoromethoxyphenylhydrazone-FCCP; 2.0 µM final concentration); Oligomycin (2.0 µM final concentration) was added to measure proton leak. The readouts were normalized to cell numbers and analyzed using Seahorse XF96 Wave software.

Many studies have shown that hiPSCs and human embryonic stem cells (hESCs) use the glycolytic pathway to maintain pluripotency [37]. Therefore, a classical glycolytic stress test was performed using the XFe96 based on the following procedure: (1) cells were cultured in unbuffered Seahorse medium without glucose and pyruvate, (2) the proton efflux rate (PER) was measured after the addition of saturating amounts of glucose, (3) oligomycin was added to inhibit ADP phosphorylation, and (4) 2-DG was added to inhibit glycolysis. The different assay parameters: basal glycolysis, compensatory glycolysis, total proton efflux, and post 2-DG acidification were normalized to cell number and analyzed using Seahorse XF96 Wave software.

The mitochondrial-derived bioenergetics health index (mitoBHI) [38], an index of mitochondrial quality was determined using the following formula: MitoBHI = LOG ((ATP production)^a^ x (Spare reserve capacity)^b^)/((Proton leak)^c^ x (non-mitochondrial respiration)^d^), where a, b,c, d exponents modify the relative weight of each respiratory parameter and by default are equivalent to 1 in this experiment. The glycolytic BHI (glycoBHI), an index of glycolytic respiration was determined using the formula: GlycoBHI = LOG ((Basal glycolysis)^a^ x (compensatory glycolysis)^b^) / ((Mito PER)^c^ x (Post 2-DG acidification)^d^)).

### Live-cell fluorescence microscopy

Fluorescence images of live cells were acquired using an EVOS FL inverted light/epifluorescence microscope with 40X/0.65 objective and a Sony ICX445 monochrome CCD digital camera. Red fluorescence from Mitotracker Red CM-H2Xros was measured using a 530 nm excitation and a 593 nm emission filter set. Blue fluorescence from Nucblue Hoechst was measured using a 360 nm excitation and a 447 nm emission filter set. All live cells were imaged on 35 mm dishes containing phenol-red-free basal medium. Image acquisition was performed one dish at a time with a maximum time of 30 min between dishes. All dishes were stored in a humidified 37 °C, 5% CO2 incubator until image acquisition. All images of live cells were taken on the same day as the labeling of mitochondria. 10–14 images were acquired per dish, and three dishes were stained per trial. Three independent trials were performed for each of the hiPSC lines used in the study.

### Mitochondrial morphology and network analysis

The images generated for the hiPSCs were pre-processed on Image-J following the steps previously outlined [39–42]. After pre-processing, the images were skeletonized. Post-skeletonization, images were segmented using Adobe Photoshop CC 2018. These segmented images were opened in Image-J software, and the MiNA macros were used to quantify mitochondrial morphological parameters of each segmented image. Since hiPSC lines have different colony morphologies, we normalized the parameters generated through MiNA by the cell surface area and the number of cells, which was also measured using Image-J.

### Statistical analysis

To ensure scientific rigor and reproducibility, for the bioenergetics analyses, an ANOVA design accounting for 3 biological and 8–12 technical replicates from control (BJ-hiPSC) and diseased (SBG6 (m.3243A>G), and SBG7-(m.14739G > A)-hiPSCs) that are nested within groups was used to identify any differences with respect to control BJ hiPSCs. Post-hoc Tukey HSD tests were used to identify differences among specific groups. Data are presented as the mean ± standard deviation (SD) and were analyzed using the GraphPad Prism 5 program (GraphPad Software, San Diego, CA, USA). A *p* < 0.05 was considered significant.

## Results

### hiPSCs are generated from reprogrammed patient fibroblasts

To investigate the ability of patient fibroblast cells with tRNA mutations in the mitochondrial genome to undergo iPSC reprogramming using an RNA approach, SBG6 (m.3243A>G), SBG7 (m.14739G > A), and BJ fibroblast cell lines were transfected with non-modified POU5F1, SOX2, KLF4, cMYC, NANOG, and LIN28 mRNAs, immune evasion E3, K3, and B18 mRNAs, and reprogramming-enhanced mature, double-stranded microRNAs from the 302/367 cluster. Fibroblasts were transfected daily for 4 days in feeder-free/xeno-free culture conditions to prevent contamination from non-human cells and biological material. After 13 and 18 days respectively, small rounded, putative hiPSC colonies began to arise that developed into well-circumscribed, compact colonies for SBG7 (m.14739G > A) and SBG6 (m.3243A>G) hiPSCs (Fig. 1). Patient cell lines did show variability in their ability to be successfully reprogrammed with SBG7 (m.14739G > A) fibroblast line reprogrammed during the first attempt to generate stable hiPSC lines, which was comparable to the control BJ line. In contrast, the SBG6 (m.3243A>G) fibroblast line underwent the reprogramming process twice before forming stable putative hiPSC lines. Once reprogrammed, the respective hiPSC lines were capable of robust expansion with comparable proliferation rates (SBG6-(m.3243A>G)- hiPSC- 26.02 h population doubling time; SBG7-(m.14739G > A)- hiPSC- 33.98 h) to the control BJ-hiPSC line (28.37 h). After stable proliferation and 12 + passages, these hiPSCs were assessed for pluripotency characteristics by immunocytochemistry and flow cytometry.

### hiPSCs express markers characteristic of pluripotent stem cells

**Fig. 2:**
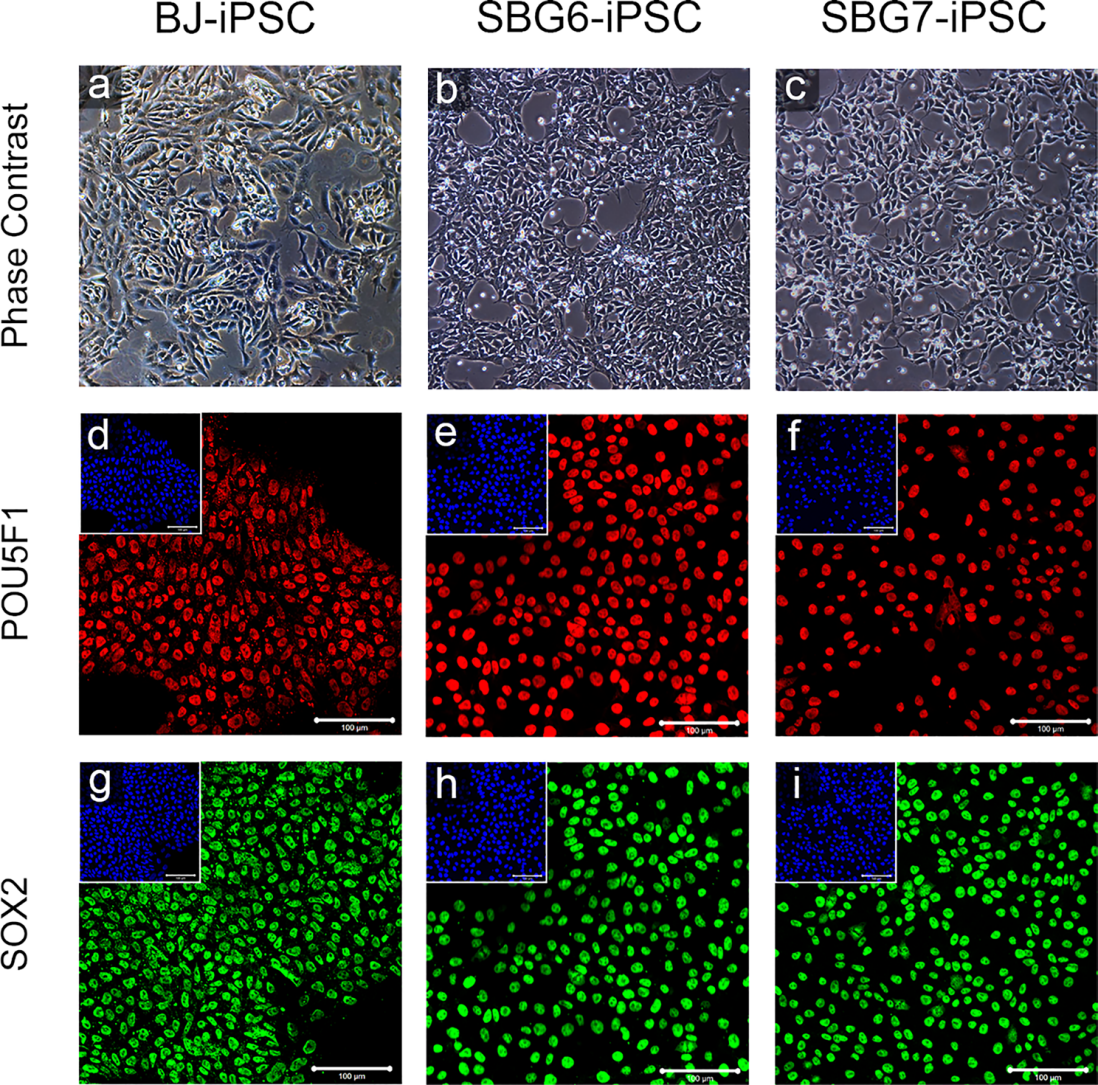
hiPSCs display classical pluripotent stem cell morphology and positively express core pluripotency transcription factors. Phase contrast images of control (**a**) BJ-hiPSC and diseased (**b**-**c**) SBG6-(m.3243A > G)-and SBG7-(m.14739G > A)-hiPSCs show the presence of pluripotent stem cell morphology with cells exhibiting a high nuclear-cytoplasmic ratio and large nucleoli, representative of actively dividing undifferentiated cells. Control BJ-hiPSC (**d**,**g**) and diseased SBG6-(m.3243A > G)-hiPSC (**e**,**h**), SBG7-(m.14739G > A)-hiPSC (**f**,**i**), are positive for POU5F1 (**d**-**f**) and SOX2 (**g**-**i**) pluripotent markers. Image inserts contain corresponding DAPI nuclear counterstain. DAPI, 4’,6-diamidino-2-phenylindole. Scale bar = 100 μm

Phase contrast images revealed that putative patient-derived SBG6-(m.3243A>G) and SBG7-(m.14739G > A) hiPSC lines displayed typical hiPSC morphology, including a high nucleus to cytoplasm ratio and large, prominent nucleoli comparable to the control BJ-hiPSC line (Fig. 2). Putative hiPSCs were assessed for the expression of pluripotency markers. Immunocytochemistry analysis of patient-derived hiPSCs with antibodies directed against POU5F1 and SOX2 demonstrated that these pluripotency transcription factor proteins were robustly expressed and correctly localized to the nucleus comparably to BJ-hiPSCs (Fig. 2). Flow cytometry analysis showed POU5F1, SOX2, and NANOG were co-expressed > 95% of analyzed control BJ-hiPSCs, 93.6% of analyzed SBG6-(m.3243A>G) hiPSCs, and 97.4% of analyzed SBG7- (m.14739G > A)- hiPSCs (Supplementary Figure. 1).

**Fig. 3:**
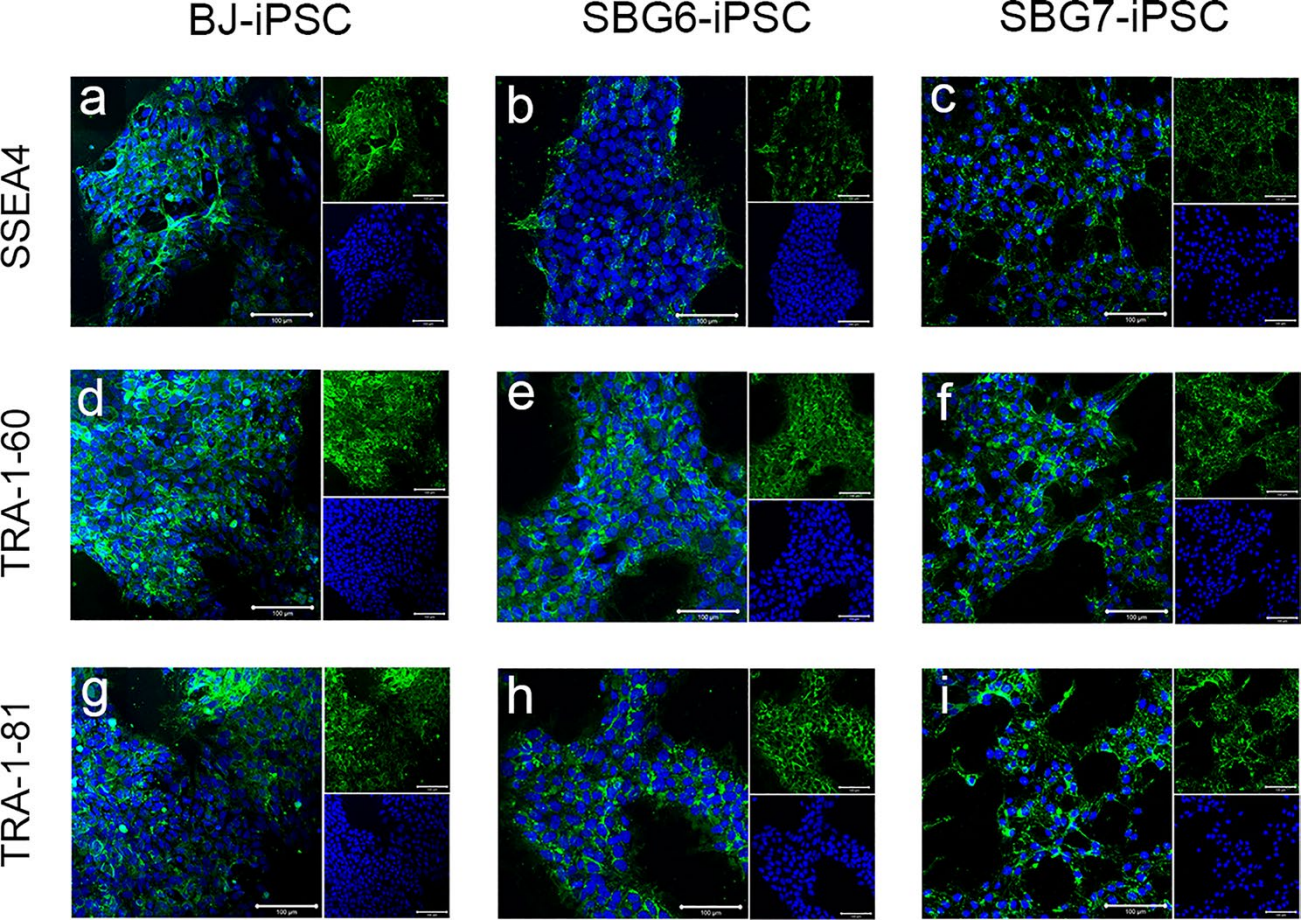
Diseased hiPSCs express the cell surface pluripotency markers SSEA4, TRA-1-60, and TRA-1-81. Control BJ-hiPSC (**a**,**d**,**g**) and diseased SBG6-(m.3243A > G)-hiPSC (**b**,**e**,**h**), SBG7-(m.14739G > A)-hiPSC (**c**,**f**,**i**), are positive for SSEA4 (**a**-**c**), TRA1-60 (**d**-**f**) and TRA-1-81 (**g**-**i**) cell surface markers. Image inserts contain corresponding DAPI nuclear counterstain. DAPI, 4’,6-diamidino-2-phenylindole. Scale bar = 100 μm

Patient-derived SBG6-(m.3243A>G) and SBG7-(m.14739G > A)- hiPSCs were also assessed for the canonical hiPSC cell surface glycoprotein and glycolipid epitopes stage-specific embryonic antigen (SSEA)-4, tumor-related antigen (TRA)-1-60, and TRA-1-81 [43–45]. Immunocytochemistry showed robust expression of SSEA-4, TRA-1-60, and TRA-1-81 in both SBG6-(m.3243A>G) and SBG7-(m.14739G > A)- hiPSCs and at comparable levels with control -BJ- hiPSCs (Fig. 3). Flow cytometry analysis showed patient-derived SBG6-(m.3243A>G) and SBG7-(m.14739G > A)- hiPSCs expressed pluripotency surface markers SSEA4, TRA-1-60, and TRA-1-81 (Supplementary Figure. 2). SSEA-4 was expressed in 94.9% of SBG6-(m.3243A>G) hiPSCs, 95.8% of SBG7-(m.14739G > A)-hiPSCs, and 98.5% of control BJ hiPSCs. TRA-1-60 was expressed in 99% of SBG6-(m.3243A>G) hiPSCs, 98.5% of SBG7-(m.14739G > A)-hiPSCs, and 96.8% of control BJ hiPSCs. TRA-1-81 was expressed in 96.5% of SBG6-(m.3243A>G)-hiPSCs, 85.1% of SBG7-(m.14739G > A)-hiPSCs, and 83.1% of control BJ hiPSCs. Taken together, immunocytochemistry and flow cytometry data indicate activation of the endogenous pluripotency network and reprogramming of mitochondrial disease patient fibroblasts into hiPSCs.

### Karyotype analysis demonstrated no aneuploidies or significant structural abnormalities

An important feature of creating hiPSCs is the ability to generate cell models with normal karyotypes indicative of a stable nuclear genome [46]. This is important so that further studies can be conducted to measure the effect of mitochondrial genome perturbations on mitochondrial function without chromosomal abnormalities confounding data interpretation or causing lethal phenotypes. In our study, karyotyping was performed three times with a minimum of 20 metaphases counted and imaged for each cell line to determine if reprogramming led to karyotypic abnormalities. At the band level analyzed (400–450), aneuploidies and significant structural chromosome abnormalities present in the cell lines can be evaluated. Our analysis indicated that no aneuploidies or significant structural abnormalities were present in both the SBG6-(m.3243A>G) and SBG7-(m.14739G > A) hiPSCs (Supplementary Fig. 3).

### Sanger sequencing demonstrates the presence of mitochondrial mutations in hiPSCs

**Fig. 4:**
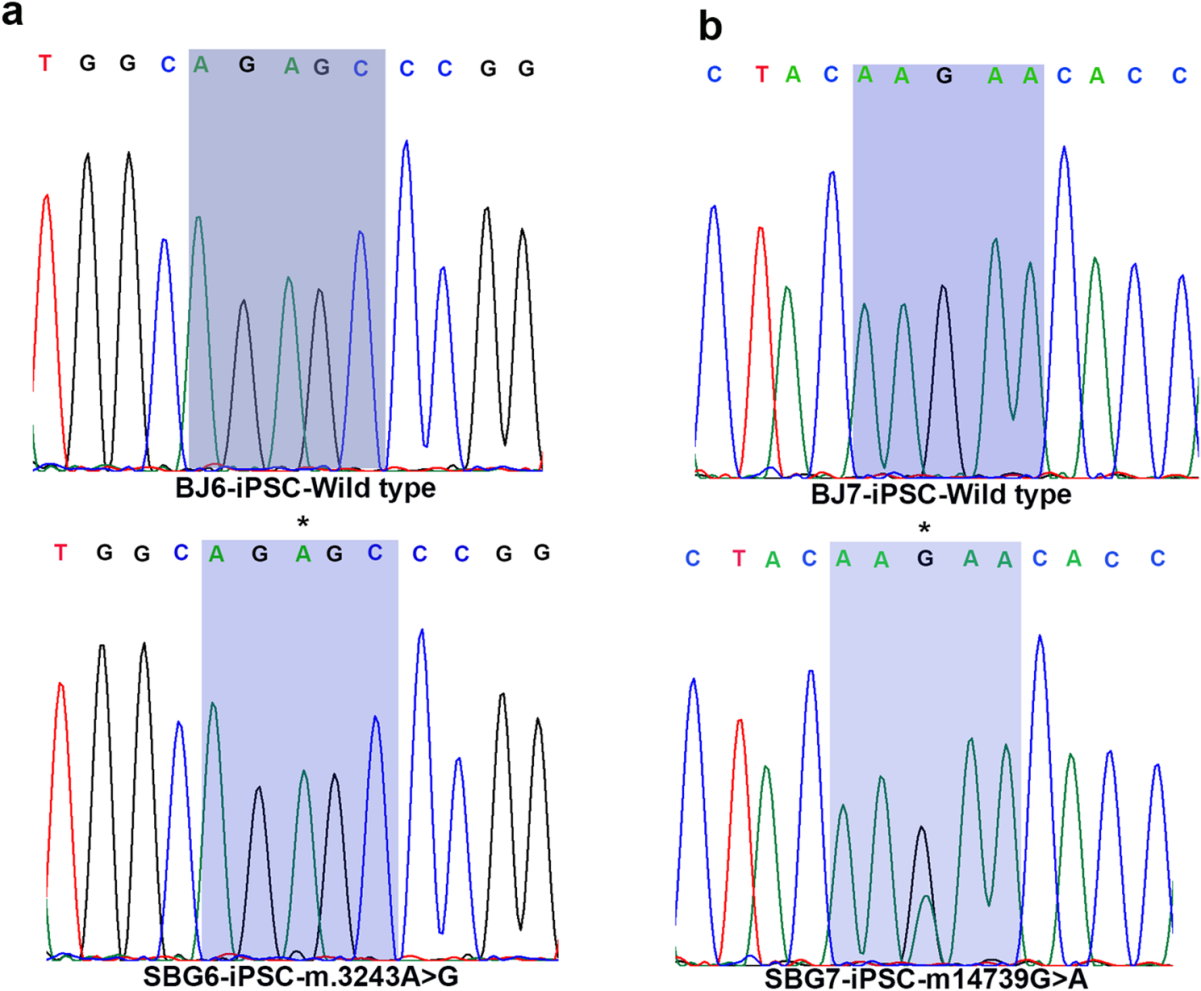
Detection of mutation through PCR amplification and Sanger sequencing of control, BJ-hiPSC and diseased SBG6-(m.3243A>G)-and SBG7-(m.14739G > A)-hiPSCs. Following extraction of mtDNA, the detection of mutation through PCR amplification and Sanger sequencing of mtDNA was conducted in (**a**) SBG6-(m.3243A>G)-hiPSC (**b**) SBG7-(m.14739G > A)-hiPSC. Mutation was detected in all diseased hiPSCs, while absent in the control, BJ-hiPSCs

To confirm the presence of the mitochondrial genome mutation in all the generated hiPSCs, the mtDNA was extracted from whole cell pellets, and specific regions of interest within the genes (for SBG6-(m.3243A>G)-hiPSCs; SBG7-(m.14739G > A)-hiPSCs) were PCR amplified using primers detailed in Supplementary Table 1. After the PCR products were purified and quantified to the appropriate specification, Sanger sequencing was necessary to confirm the presence of the disease-causing mutation after reprogramming. The results demonstrate that the m.3243A>G mutation is present in SBG6-(m.3243A>G) –hiPSC line (Fig. 4a) and the m.14739G > A mutation is present in the SBG7-(m.14739G > A)-hiPSC line (Fig. 4b). The control BJ-hiPSC samples were found to be devoid of these specific point mutations.

### Mitochondrial morphological analysis indicates the presence of highly elongated to hyperfused mitochondria in generated hiPSCs

**Fig. 5:**
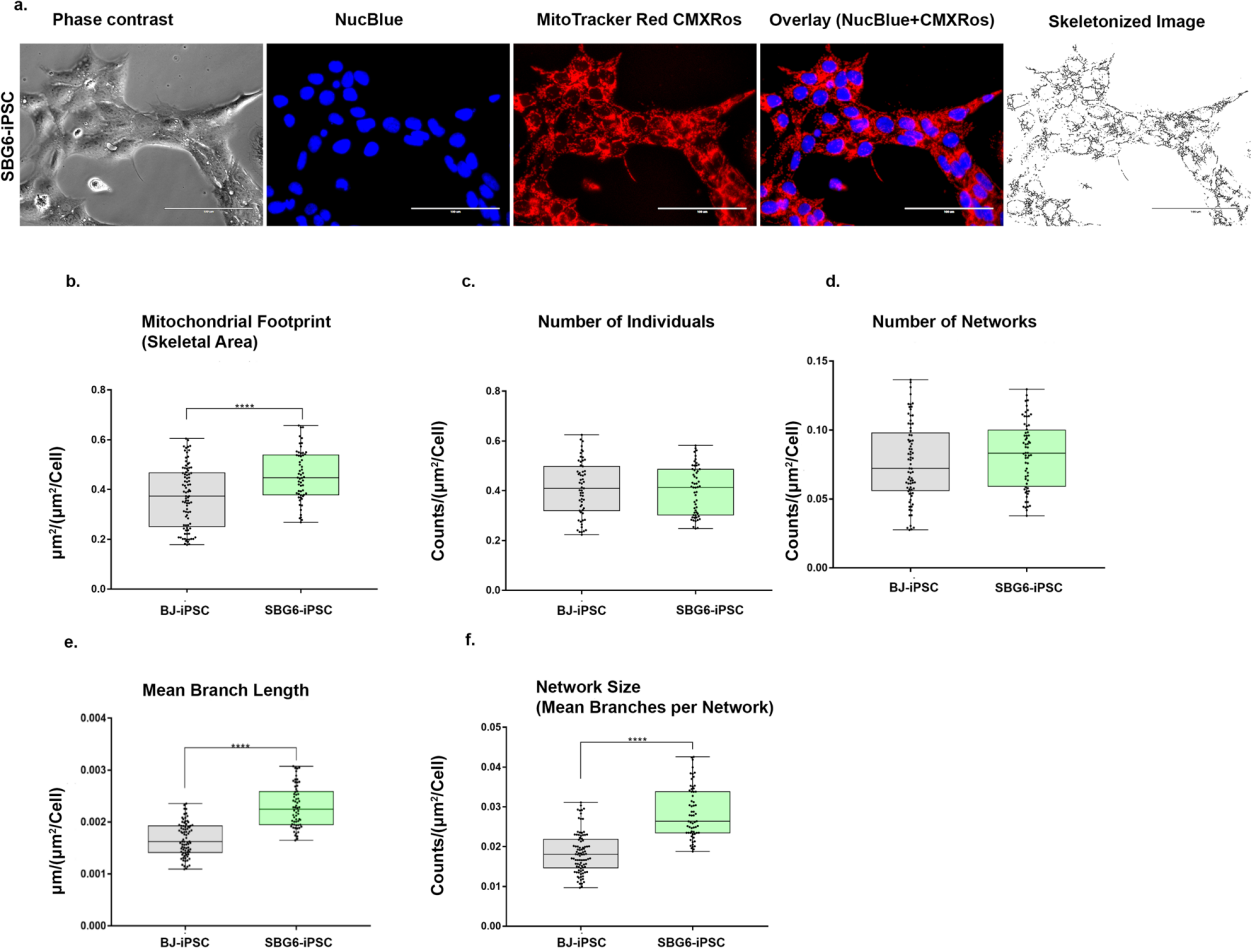
Mitochondrial morphology of SBG6-(m.3243A>G) hiPSC. (**a**) Representative images of SBG6-hiPSC lines stained with MTR. Phase contrast, NucBlue (nucleus), MitoTracker Red, overlay and skeletonized images of SBG6-(m.3243A>G) hiPSC. Morphology parameters include (**b**) mitochondrial footprint (**c**) number of individuals (**d**) number of networks (**e**) mean branch length and (**f**) network size. All data are representative of 10–14 images taken from three independent dishes for each cell line. The bars represent minimum and maximum values, and each black dot represents different data points. **** p < 0.0001, Scale bar = 100 μm

**Fig. 6:**
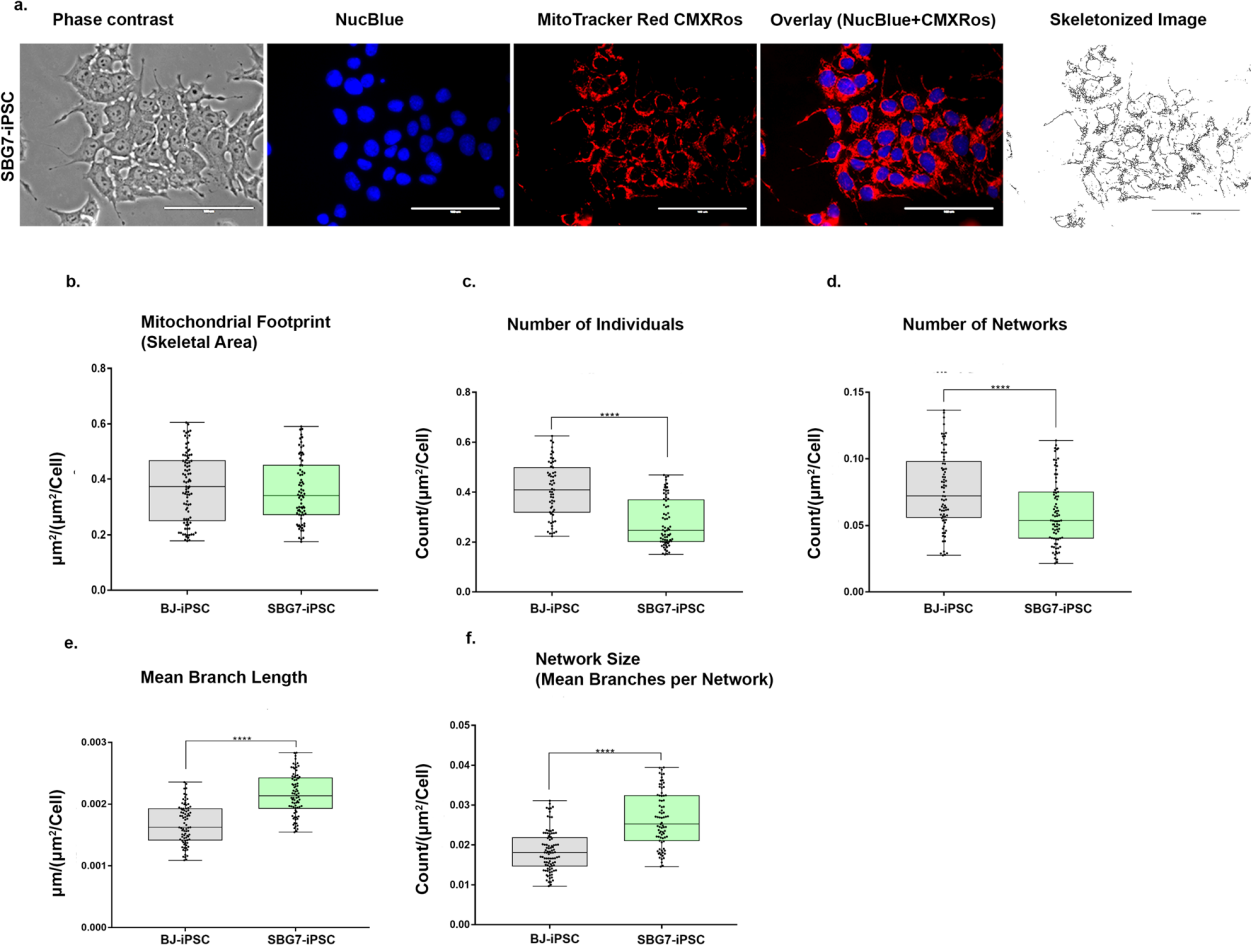
Mitochondrial morphology of SBG7-(m.14739G > A)-hiPSC. (**a**) Representative images of SBG7-(m.14739G > A)-hiPSC lines stained with MTR. Phase contrast, NucBlue (nucleus), MitoTracker Red, overlay and skeletonized images of SBG7-(m.14739G > A)-hiPSC. Morphology parameters include (**b**) mitochondrial footprint (**c**) number of individuals (**d**) number of networks (**e**) mean branch length and (**f**) network size. All data are representative of 10–14 images taken from three independent dishes for each cell line. The bars represent minimum and maximum values, and each black dot represents different data points. **** *p* < 0.0001, Scale bar = 100 μm

In a recent study, we used Mitochondrial Network Analysis (MiNA) toolset to quantify the different mitochondrial morphologies in SBG6 (m.3243A>G) and SBG7 (m.14739G > A) parental fibroblasts [40] used for reprogramming in this study. Results demonstrated the presence of sparse, fragmented mitochondria in the SBG6 (m.3243A>G) fibroblast cell line and fused mitochondria in the SBG7 (m.14739G > A) fibroblast cell line compared to the control BJ fibroblast line [40]. These results suggested perturbations in mitochondrial morphology in the two parental fibroblast lines containing different tRNA mutations. We then examined mitochondrial morphology in the SBG6 (m.3243A>G)- hiPSC and SBG7-(m.14739G > A) -hiPSC and compared them with the control BJ-hiPSCs. Previous studies have demonstrated that interactions between bases in tRNA are very important for maintaining the tertiary structure of mitochondria, with mutations causing structural alterations, impairment of the mitochondrial translation machinery, and a decrease in overall mitochondrial respiration [47]. The degree of mitochondrial fragmentation, branching, or elongation was assessed by measuring the number of individuals, the number of networks, network size, and the mean branch length of the network (Supplementary Fig. 4). The lower number of individuals with a longer branch length is consistent with a more elongated mitochondrial network. Both SBG6-(m.3243A>G) and SBG7-(m.14739G > A)- hiPSCs exhibited a significantly higher mean branch length (*p < 0.001*) (Figs. 5e and 6e), and significantly higher network size (Figs. 5f and 6f) relative to the control BJ-hiPSC lines. While both SBG6-(m.3243A>G) and SBG7-(m.14739G > A)-hiPSCs exhibited a decrease in the number of individuals (Figs. 5c and 6c), SBG7-(m.14739G > A)-hiPSCs exhibited a significantly lower number of individuals and networks (Fig. 6c and d) (*p < 0.001*), relative to the control BJ-hiPSC line.

### Metabolic comparison of oxidative phosphorylation of BJ-hiPSCs, SBG6-(m.3243A>G) and SBG7-(m.14739G > A)-hiPSCs

**Fig. 7:**
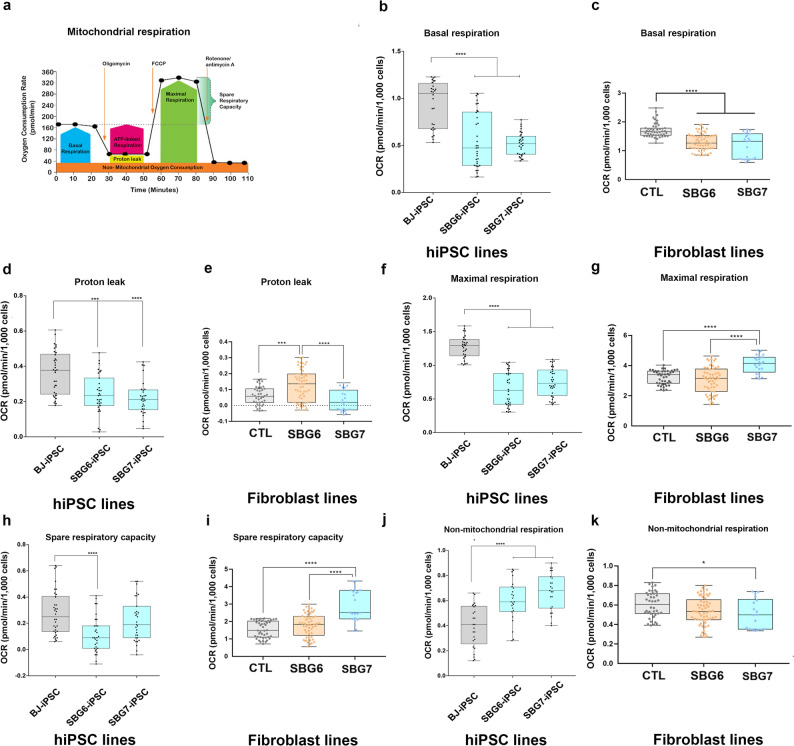
Mitochondrial respiratory profile of hiPSC and parental fibroblast cell lines. (**a**) Scheme of expected oxygen consumption rate (OCR) under basal conditions; (**b**,**d**,**f**,**h**,**j**)- OCR profile of control BJ-iPSC and diseased SBG6-(m.3243A>G)-and SBG7-(m.14739G > A)-hiPSCs; (**c**,**e**,**g**,**i**,**k**)- OCR profile of control BJ-fibroblasts and diseased SBG6-(m.3243A>G)-and SBG7-(m.14739G > A) fibroblasts; (**b**,**c**) basal respiration; (**d**,**e**) proton leak; (**f**,**g**) maximal respiration; (**h**,**i**) spare respiratory capacity after FCCP injection; (**j**,**k**) non mitochondrial respiration after Rot/AA injection. All parameters are in pmol/min/1000 cells. Data are mean +/- SD. Experiments were repeated at least three times on different days under the same conditions. *p < 0.05 **p < 0.01 ***p < 0.001 ****p < 0.00001. Comparative analyses for all diseased (SBG6-(m.3243A>G), SBG7-(m.14739G > A)) hiPSCs were conducted with the control BJ-hiPSC line; while comparative analyses for all diseased (SBG6-(m.3243A>G), SBG7-(m.14739G > A)) fibroblasts were conducted with the control BJ-fibroblast line

Active measurements and tracing of cell respiratory control provide a comprehensive assessment of mitochondrial function in a cellular population and highlight mitochondrial dysfunction. In a single experimental run, several mitochondrial functional parameters are measured by the addition of mitochondrial inhibitors and uncouplers. The generic mitochondrial respiration profile via measurement of oxygen consumption rate (OCR) in a normal functioning cell population that is subject to the mito-stress test is detailed in Fig. 7a. Briefly, the purpose of the test is to measure respiration under basal conditions and subsequently expose the cells to several stressors, such as sequential addition of oligomycin (ATP synthase inhibitor) that allows for measurement of OCR resulting from non-mitochondrial respiration and proton leak; Carbonyl cyanide-p-trifluoromethoxyphenylhydrazone- FCCP (uncoupler) that permits measurement of the maximum state of mitochondrial respiration via the ETC independent of ADP phosphorylation; and finally rotenone and antimycin A (complex I and III inhibitors respectively) which completely block the respiratory ETC leading to the ability to measure non-mitochondrial background and thus calculate mitochondrial respiration. Results indicate that, similar to previously published studies [37, 48, 49], the control BJ-hiPSCs demonstrate a lower response to oligomycin and FCCP when compared with the parental BJ-fibroblast which may indicate decrease in spare capacity of these cells (Fig. 7h and i) with a relatively high proton leak (Fig. 7d and e), upon reprogramming of the BJ-fibroblasts to BJ-hiPSCs.

Based on OXPHOS analysis, the diseased SBG6-(m.3243A>G), SBG7(m.14739G > A)- hiPSCs demonstrated lower mitochondrial dependency than the control BJ-hiPSC line. Results indicate that the diseased SBG6-(m.3243A>G) and SBG7-(m.14739G > A) hiPSCs exhibited reduced basal respiration when compared with BJ-hiPSCs, with statistically significant reduced respiration in SBG6-(m.3243A>G)-hiPSC (42% decrease; *p* = 0.00001) and SBG7-(m.14739G > A) hiPSC (45% decrease; *p* = 0.0001) (Fig. 7b). Similarly, the diseased SBG6-(m.3243A>G) and SBG7-(m.14739G > A) fibroblasts exhibited reduced basal respiration when compared with BJ-fibroblasts, with statistically significant reduced respiration in SBG6-(m.3243A>G) (23% decrease; *p* = 0.00001) and SBG7-(m.14739G > A) (32% decrease; *p* = 0.0001) fibroblasts (Fig. 7c). Both SBG6-(m.3243A>G) and SBG7-(m.14739G > A) hiPSCs exhibited reduced proton leak when compared with BJ-hiPSCs with statistically significant reduced proton leak in SBG6-(m.3243A>G) -hiPSC (33% decrease; *p* = 0.001) and SBG7-(m.14739G > A)-hiPSC (40% decrease; *p* = 0.00001) (Fig. 7d). However, the diseased SBG6-(m.3243A>G) fibroblast exhibited increased proton leak when compared with BJ-fibroblasts (106% increase; *p* = 0.0001), while SBG7-(m.14739G > A) fibroblast exhibited a reduced proton leak (54% decrease) when compared with control BJ-fibroblasts (Fig. 7e**).**

However, the maximal respiration was significantly reduced in all diseased lines (SBG6-(m.3243A>G) hiPSC- 49% decrease; *p* = 0.00001; SBG7-(m.14739G > A)-hiPSC- 41% decrease; *p* = 0.00001; when compared with control BJ-hiPSCs (Fig. 7f). In line with reduced maximal respiration, the spare respiratory capacity was reduced in both SBG6-(m.3243A>G) -hiPSC (59% decrease; *p* = 0.00001) and SBG7-(m.14739G > A)-hiPSC (20% decrease), when compared with the control BJ-hiPSC (Fig. 7h). The trajectory for maximal respiration and spare respiratory capacity was similar for each of the fibroblast cell lines analyzed, with SBG7-(m.14739G > A)-fibroblast exhibiting statistically significant increase in maximal respiration (27% increase, *p* = 0.00001) (Fig. 7g), with a statistically significant increase in spare reserve capacity (90% increase, *p* = 0.00001) (Fig. 7i), relative to control BJ-fibroblasts. However, the maximal respiration and spare reserve capacity measurements for SBG6-(m.3243A>G) -fibroblasts were not significantly different from the control BJ-fibroblasts, although a slight decrease (6%) in maximal respiration (Fig. 7g) and a slight increase (16%) in spare reserve capacity (Fig. 7i) were observed. The non-mitochondrial respiration was significantly increased in SBG6-(m.3243A>G) -hiPSC (51% increase; *p* = 0.00001) and SBG7-(m.14739G > A)-hiPSC (70% increase; *p* = 0.0001) when compared with control BJ-hiPSC (Fig. 7j), while it was reduced (11%) in SBG6-(m.3243A>G) fibroblast and significantly reduced (16%, *p* = 0.05) in SBG7-(m.14739G > A)-fibroblast when compared with control BJ fibroblasts. (Fig. 7k).

### Metabolic comparison of glycolytic profile of BJ-hiPSCs, SBG6-(m.3243A>G) and SBG7-(m.14739G > A)-hiPSCs

**Fig. 8:**
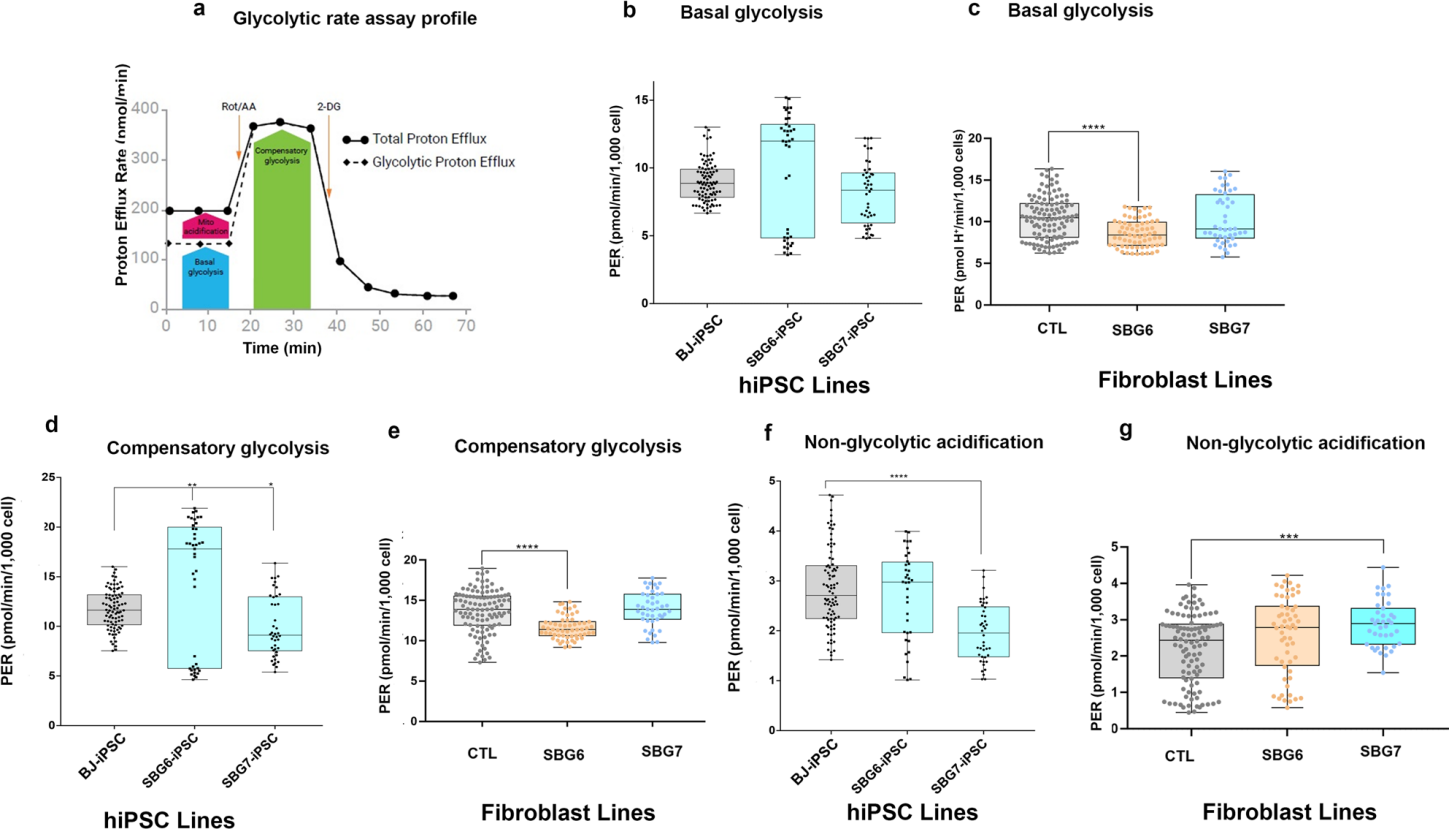
Glycolytic profile of hiPSC and parental fibroblast cell lines. (**a**) scheme shows the glycolytic acidification, (**b**,**d**,**f**) PER of control BJ-iPSC and diseased SBG6-(m.3243A>G)-and SBG7-(m.14739G > A)-hiPSCs; (**c**,**e**,**g**) PER of control BJ-fibroblast and diseased SBG6-(m.3243A>G)-and SBG7-(m.14739G > A)- fibroblasts; (**b**,**c**) basal glycolysis; (**d**,**e**) compensatory glycolysis after ETC blocking using Rot/AA; (**f**,**g**) post 2-DG (non-glycolytic acidification). Data are shown in pmol H+/min/1000 cells as mean +/- SD. Experiments were repeated at least three times in different days under the same conditions. *p < 0.05 **p < 0.01 ***p < 0.001 ****p < 0.00001. Comparative analyses for all diseased (SBG6-(m.3243A>G), SBG7-(m.14739G > A)) hiPSCs were conducted with the control BJ-hiPSC line; while comparative analyses for all diseased (SBG6-(m.3243A>G), SBG7-(m.14739G > A)) fibroblasts were conducted with the control BJ-fibroblast line. PER: proton efflux rate

The preference of glycolysis over OXPHOS under both normoxic and hypoxic conditions is a key characteristic of hiPSCs [37]. Glycolysis measurements using the extracellular flux analyzer provide readouts of proton efflux rate (PER). Concurrent addition of the ETC inhibitors rotenone and antimycin A permits PER measurements in basal glycolysis, while inhibition of glycolysis by 2, deoxy-glucose (2-DG) allows measurement of other parameters like compensatory glycolysis, total proton efflux, and post 2-DG acidification (Fig. 8a). Although both SBG6-(m.3243A>G) and SBG7-(m.14739G > A)-hiPSCs exhibited overall similar respiration profiles, the glycolytic profile was different for each line when compared with the control BJ-hiPSCs (Fig. 8b), with a different compensatory mechanism for each cell line (Fig. 8d); indicating the possibility that the reprogrammed hiPSCs are potentially affected by the presence of the mutation and also by their original physiological environment and epigenetic memory of the respective parental patient fibroblasts. SBG6-(m.3243A>G) -hiPSCs exhibited an increase (10%), while SBG7-(m.14739G > A)-hiPSCs exhibited a decrease (10%) in basal glycolysis relative to control BJ-hiPSCs (Fig. 8b). However, SBG6-(m.3243A>G) fibroblasts exhibited a 17% decrease (*p = 0.0001*), while SBG7-(m.14739G > A) fibroblasts exhibited a 1% decrease in basal glycolysis relative to control BJ fibroblasts (Fig. 8c**)**. Similarly, SBG6-(m.3243A>G)-hiPSCs exhibited an increase (22%, *p* = 0.001), while SBG7-(m.14739G > A)-hiPSCs exhibited a decrease (15%) in compensatory glycolysis relative to control BJ-hiPSC (Fig. 8d). However, SBG6-(m.3243A>G) fibroblasts exhibited a 15% decrease (*p = 0.0001*), while SBG7 (m.14739G > A) fibroblasts exhibited a 1% increase in compensatory glycolysis relative to control BJ fibroblasts (Fig. 8e). The observed increase in basal and compensatory glycolysis in SBG6-(m.3243A>G) -hiPSCs is potentially a compensation mechanism in response to reduced mitochondrial oxygen consumption and indicates that SBG6-(m.3243A>G) -hiPSC is dependent more on glycolysis than on OXPHOS, while SBG7-(m.14739G > A)-hiPSC is dependent on both OXPHOS and glycolysis pathways. Non-glycolytic acidification after inhibition of glycolysis using 2-DG showed no statistically significant decrease (5%) in SBG6-(m.3243A>G) -hiPSC, while demonstrating a statistically significant decrease in SBG7-(m.14739G > A)-hiPSC (31% decrease, *p* = 0.00001) when compared with control BJ-hiPSC (Fig. 8f). However, non-glycolytic acidification showed no statistically significant increase (16%) in SBG6-(m.3243A>G) -fibroblasts, while demonstrating a statistically significant increase in SBG7-(m.14739G > A)-fibroblasts (30%, *p* = 0.0001) when compared with control BJ-fibroblasts (Fig. 8g).

## Discussion

Two hiPSC lines with specific mitochondrial tRNA mutations (m.3243A>G, m.14739G > A) were generated and characterized for their mitochondrial dysfunction to better understand the cellular physiology associated with these mitochondrial diseases early on in development and can be used to identify novel therapeutics. Similar to previously published studies [20, 25, 28, 50, 51], the two hiPSCs exhibited many classic hallmarks of a pluripotent stem cell. Results are in concordance with our earlier study, which demonstrated the derivation and characterization of a karyotypically stable non-viral hiPSC line reprogrammed from a mitochondrial disease patient fibroblast [20]. Sanger sequencing confirmed the presence of the mutation in all reprogrammed hiPSCs, and is in line with previous studies that have demonstrated the potential for reprogramming to generate hiPSCs with low percentages of pathogenic mtDNA [24, 28, 30]. Generating additional hiPSCs with disease-specific tRNA mutations is an essential step for better understanding mitochondrial disorders during early development and in patient populations. These patient-specific hiPSCs represent a stable source of cells for further differentiation into specialized cell types like neurons and muscles. Investigating the differences between cell lines and tissues will be extremely important in further understanding the complexities associated with mitochondrial diseases with different tRNA mutations.

During reprogramming of fibroblasts and generation of hiPSCs, dynamic mitochondrial morphology changes were observed that were in line with changes in energy metabolism [52–56]. For the first time, we have conducted a comprehensive quantification of mitochondrial dynamics in hiPSCs with pathogenic tRNA mutations in the mtDNA. Using the MiNA toolkit with ImageJ, we were able to quantify the different morphologies in both the SBG6-(m.3243A>G) and (SBG7-(m.14739G > A) hiPSCs. We hypothesized that both the hiPSCs would contain sparse, fragmented, and immature mitochondria, as previously observed in hiPSCs [57]. However, the MiNA toolkit was sensitive enough to detect sparse mitochondria as well as mitochondria with elongated branches, indicating a tendency towards a hyperfused state. The presence of the hyperfused state is an indication of abnormal mitochondrial phenotypes [58], which could indicate that the diseased hiPSCs exhibit lower ATP production that may affect their proliferation and self-renewal potential [57]. With the presence of abnormal mitochondrial morphologies, we then postulated that the diseased hiPSCs would exhibit respiratory dysfunction.

We conducted a thorough analysis of mitochondrial function by evaluating a range of bioenergetic parameters. Valuable insights into the energy metabolism of the hiPSCs can be obtained via measurement of the OCR and PER. By using the XFe96 extracellular flux analyzer, we were able to measure the OCR as an indicator for mitochondrial respiration and PER for glycolysis. By using different mitochondrial inhibitors, the bioenergetic profile of the generated hiPSCs were analyzed for different parameters, including basal respiration, proton leak, maximal respiration, non-mitochondrial respiration, and spare respiratory capacity (SRC). It is widely acknowledged that two major pathways are involved in cellular ATP production: [1] Glycolysis, the cell’s pathway to make ATP in the absence of oxygen, and OXPHOS, the cellular pathway of ATP generation in the presence of oxygen. Indeed, bioenergetic analyses have shown that in comparison to differentiated cells, human pluripotent stem cells (hPSCs), which include hESCs and hiPSCs demonstrate a preference for glycolysis over OXPHOS [49, 53, 55, 59, 60]. Overall, our results demonstrate that both control BJ and diseased SBG6-(3243A>G) and (SBG7-(14739G > A) hiPSCs have a greater reliance on glycolysis (Fig. 8b) than mitochondrial respiration (Fig. 7b), which is in line with energy metabolism exhibited by hPSCs [49]. More importantly, we demonstrate that despite the fact that control and diseased hiPSCs rely on glycolysis, the different hiPSCs generated from patient fibroblasts are not identical in terms of oxygen consumption and basal glycolysis rates.

In the parental SBG6-(m.3243A>G) fibroblast, we hypothesized that the high proton leak and increased SRC values were a contributing factor in the cell’s decision to compensate for the lowered mitochondrial ATP rate by activating the glycolytic pathway. SRC represents the mitochondrial capacity to meet extra energy requirements, beyond the basal level, in response to different cellular stress events and thereby avoiding an ATP crisis [49, 61, 62]. In support of our hypothesis, despite undetectable heteroplasmy levels, SBG6-(m.3243A>G) fibroblast cells exhibited an overall lowered bioenergetic profile of low basal and maximal respiration, as well as higher SRC values when compared with control BJ-fibroblasts. Studies on mouse myocytes and iPSC-derived myocytes further support the importance of SRC in cell survival [63]. In the reprogrammed SBG6-(m.3243A>G) hiPSC line, we observed an overall decrease in basal respiration and SRC values, which could be attributed to high levels of glycolysis seen in this cell line when compared with control BJ-hiPSCs.

A previous clinical study identifying the m.14793G > A mutation revealed a combined complex I, III and IV deficiency with a 29% mutation burden in fibroblasts [19]. Using the same fibroblast line carrying the m.14793G > A mutation revealed 34% mutation burden with no change in electron transport activity (complex I, III and V) when compared with control BJ-fibroblasts. Further bioenergetics analyses of the SBG7- m.14793G > A fibroblasts revealed low basal respiration, reduced proton leak, higher maximal respiration, higher spare reserve capacity, lower non-mitochondrial respiration, reduced basal glycolysis, reduced compensatory glycolysis, and increased non-glycolytic acidification when compared with control BJ-fibroblasts. Upon reprogramming, the novel SBG7-m.14793G > A hiPSC line exhibited low basal respiration, low proton leak, low maximal respiration, reduced spare reserve capacity, higher non-mitochondrial respiration, reduced basal glycolysis, reduced compensatory glycolysis, and reduced non-glycolytic acidification when compared with control BJ-hiPSCs, indicating a functional mitochondrial deficiency early on in development.

To better understand the relationship between the cellular markers for mitochondrial dysfunction and disease severity, the “bioenergetics health index or BHI” has been proposed as a biomarker for measuring overall mitochondrial dysfunction [50, 64]. The BHI value, based on the different mitochondrial bioenergetics parameters, captures positive aspects of bioenergetics function (spare reserve capacity and ATP-linked respiration) and contrasts these with potentially negative aspects (non-mitochondrial respiration and proton leak) of bioenergetics function. In this study, for the first time, we have calculated the ‘mitoBHI’ in hiPSCs, based on mitochondrial parameters, in order to better understand whether the dysfunction was apparent in the early stages of development in our hiPSC models. Individual ‘mitoBHI’ values as per the formula detailed in the methods section was lowest for SBG6-(m.3243A > G)-hiPSC at -0.59, followed by SBG7-(m.14739G > A)-hiPSC at -0.33, while the control BJ-hiPSC was estimated to be 0.05. Since the results obtained with both SBG6-(m.3243A > G)-and SBG7-(m.14739G > A)-hiPSCs demonstrated a reliance on glycolysis, we also determined the ‘glycoBHI’. Similar to the mitoBHI, the ‘glycoBHI’ captures positive aspects of glycolysis (Basal glycolysis, compensatory glycolysis) and contrasts these with potentially negative aspects of glycolysis (mitoPER and post 2DG-acidification). Individual ‘glycoBHI’ values as per formula detailed in the methods section was lowest for SBG6-(m.3243A > G)-hiPSC at 1.74, followed by SBG7-(m.14739G > A)-hiPSC at 2.00, while the control BJ-hiPSC was estimated to be 1.89. Since both SBG6-(m.3243A > G)-and SBG7-(m.14739G > A)-hiPSCs were dependent on both OXPHOS and glycolysis, we also computed the composite BHI ratio (‘mitoBHI’/’glycoBHI’) for each line. This allows us to evaluate the overall ability of the cell to meet energy demand due to its diseased state. We estimated the values to be -0.3381 for SBG6-hiPSC, -0.1623 for SBG7-hiPSC, and 0.0243 for control BJ-hiPSC. With the value for BJ-hiPSC set at 100 (normal), the composite BHI values were − 667 for SBG7-(m.14739G > A)-hiPSC and − 1389 for SBG6-(m.3243A>G)-hiPSC respectively. The observed composite BHI values thus clearly indicate that mitochondrial functional defects were severe in both the hiPSCs exhibiting tRNA mutations.

The clinical spectrum of patients carrying tRNA mutations is highly variable [65, 66]. Therefore, there is an unmet need to generate more patient-specific hiPSCs carrying pathogenic tRNA mutations to understand the impact of the tRNA mutations on mitochondrial energy metabolism during early development. Our study represents an important first step towards creating and comparing comprehensive bioenergetic analyses in two hiPSCs (SBG6-(m.3243A>G)-and SBG7-(m.14739G > A)) with control BJ-hiPSCs. Future studies will enable a better understanding of the role of pathogenic mtDNA tRNA mutations in high-energy-consuming tissues such as the brain, heart, and muscle that have not undergone rigorous systematic evaluation. Thus, use of patient-derived hiPSCs and controlled directed differentiation into specialized cell and tissue types will contribute to a better understanding of genotype, phenotype, and clinical variability associated with mitochondrial disorders in children with atypical symptoms such as growth retardation, loss of appetite or even mild exercise intolerance.

## Conclusions

In summary, we have established two hiPSC lines for disease modeling of rare childhood mitochondrial diseases exhibiting pathogenic tRNA mutations. Our results indicate that spare reserve capacity is an important biomarker for the ability of the cell to survive and adapt to high ATP demand during stress or diseased states. For the first time, the study also demonstrated that composite BHI is a good measure of mitochondrial dysfunction in hiPSCs carrying pathogenic mtDNA mutations that exhibit abnormal mitochondrial morphologies. The established patient-derived hiPSCs can be used for disease modeling in vitro to improve our understanding of the specific role of tRNA mutations on early developmental deficits, the specific role of the mitochondria in patient diagnosis and prognosis, and the evaluation of novel therapies for mitochondrial disorders.

## Supplementary Information

Below is the link to the electronic supplementary material.

Supplementary Material 1: Figure 1. Mitochondrial disease patient hiPSCs co-express pluripotency markers POU5F1, SOX2, and NANOG. Flow cytometry analysis for core pluripotency markers indicate positive co-expression of SOX2 & POU5F1 in both control BJ-iPSC (b) and diseased SBG6-(m.3243A>G)-and SBG7-(m.14739G>A)-hiPSCs (c-d). Flow cytometry also confirmed co-expression of NANOG & POU5F1 in both control BJ-iPSC (f) and in diseased SBG6-(m.3243A>G)-and SBG7-(m.14739G>A)-hiPSCs (g-h).

Supplementary Material 2: Figure 2. Flow cytometry shows mitochondrial diseased iPSCs express pluripotency glycoprotein and glycolipid epitopes. Flow cytometry analysis for cell surface pluripotency markers indicate expression for SSEA4 (a-c), TRA1-60 (d-f), TRA1-81 (g-i), in both control BJ-iPSC (a,d,g) and in diseased SBG6 (m.3243A>G) (b,e,h), SBG7 (m.14739G>A) (c,f,i) -hiPSCs.

Supplementary Material 3: Figure 3. Karyotype analysis demonstrated no aneuploidies or significant DNA structural abnormalities. Normal karyotype exhibited by (a) CTL-BJ-hiPSC (46, XY); (b) (SBG6-(m.3243A>G) hiPSC- 46, XX); (c) (SBG7-(m.14739G>A) hiPSC- 46, XX).

Supplementary Material 4: Figure 4. Mitochondrial morphology descriptors. Representative images of control BJ-hiPSC lines stained with MTR. Phase contrast, NucBlue (nucleus), MitoTracker Red, overlay and skeletonized images control BJ-hiPSC. The mitochondrial morphology is classified as (1) individuals (1) or (2) networks. Other specific morphological structures include (3) Branch length (4) junction, which contains branches and networks. Scale bar = 100 μm.

Supplementary Material 5

## Data Availability

All the data supporting the results can be found in this manuscript and the supplemental data. Please contact the corresponding author if materials generated from the current study are reasonably required.

## Abbreviations

hiPSC: Human induced pluripotent stem cell
hESC: Human embryonic stem cell
MELAS: mitochondrial encephalomyopathy, lactic acidosis, and stroke-like episodes
OXPHOS: Oxidative phosphorylation
mtDNA: Mitochondrial DNA
tRNA: transfer RNA
FCCP: 2-[2-[4-(trifluoromethoxy)phenyl]hydrazinylidene]-propanedinitrile
2DG-2: Deoxy-D-glucose
Rot: Rotenone
AA: Antimycin A
ETC: Electron Transport Chain
