# Supplementary figures and images for: Impaired mitochondrial morphology and respiratory dysfunction in human induced pluripotent stem cells with mitochondrial tRNA mutations (m.3243A>G and m.14739G>A)
Source: Orphanet J Rare Dis. 2026 Jan 29;21:73. doi: 10.1186/s13023-026-04201-z (PMC12924315; doi:10.1186/s13023-026-04201-z)

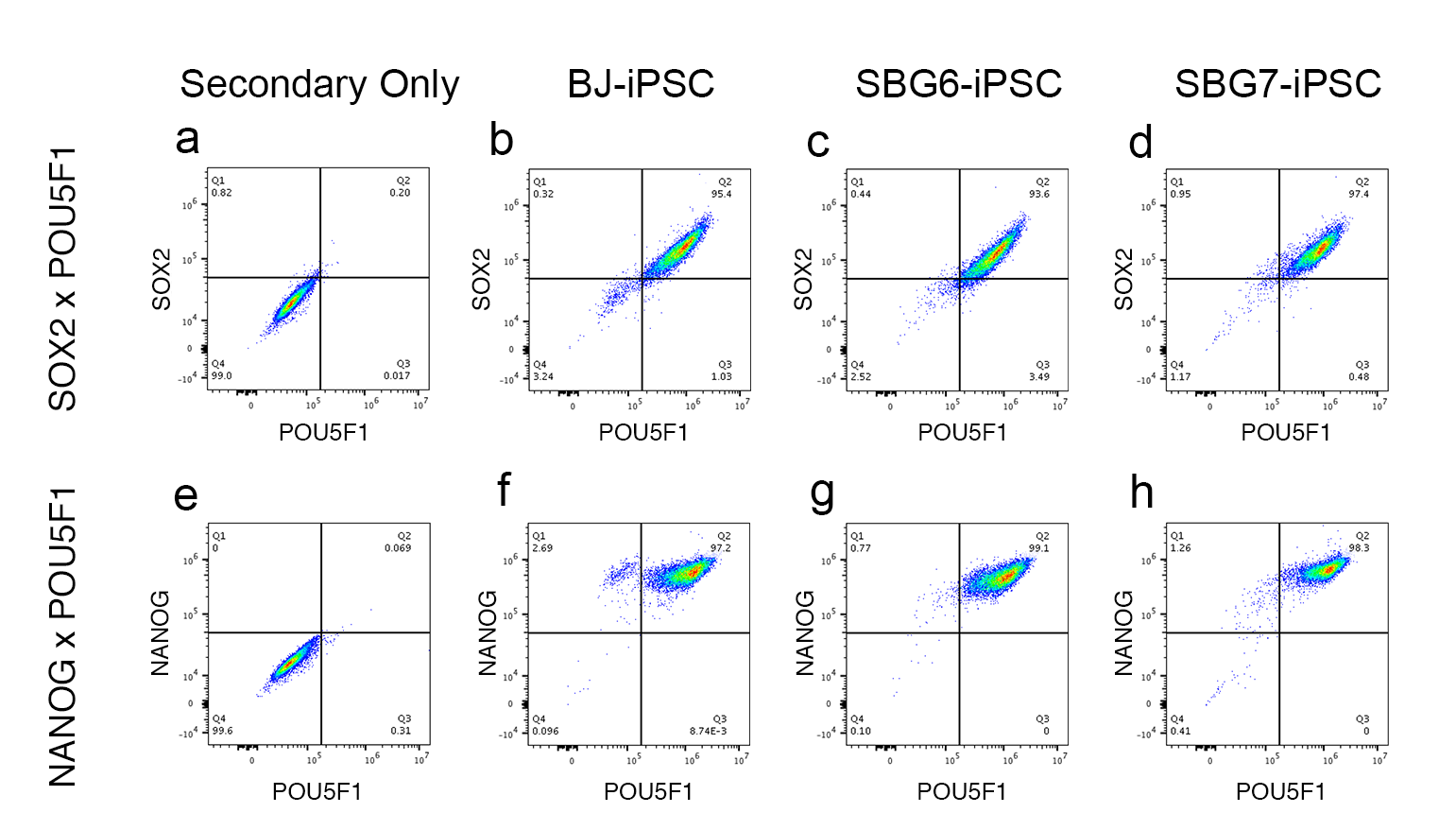

Supplement: Supplementary file 1 — Supplementary Material 1: Figure 1. Mitochondrial disease patient hiPSCs co-express pluripotency markers POU5F1, SOX2, and NANOG. Flow cytometry analysis for core pluripotency markers indicate positive co-expression of SOX2 & POU5F1 in both control BJ-iPSC (b) and diseased SBG6-(m.3243A>G)-and SBG7-(m.14739G>A)-hiPSCs (c-d). Flow cytometry also confirmed co-expression of NANOG & POU5F1 in both control BJ-iPSC (f) and in diseased SBG6-(m.3243A>G)-and SBG7-(m.14739G>A)-hiPSCs (g-h). [file 13023_2026_4201_MOESM1_ESM.tif]

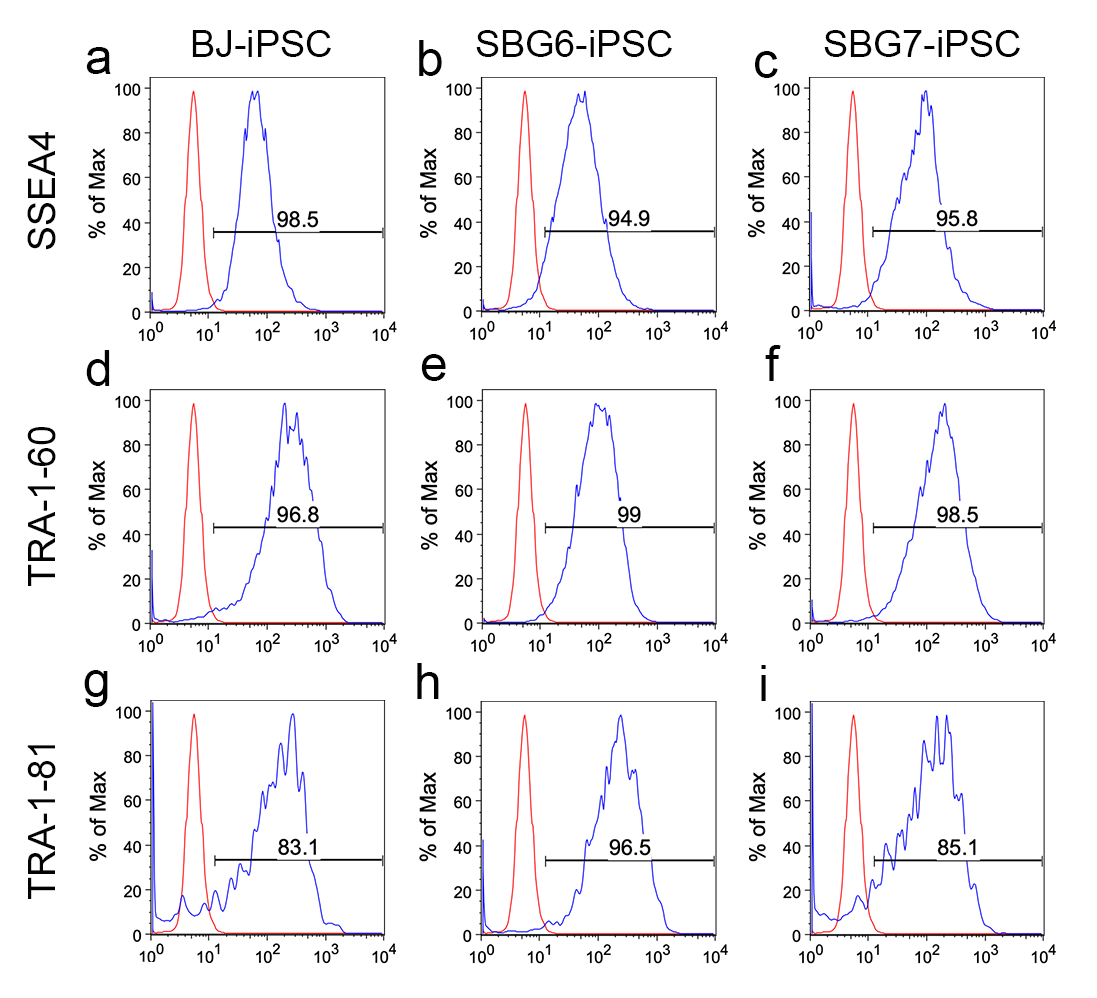

Supplement: Supplementary file 2 — Supplementary Material 2: Figure 2. Flow cytometry shows mitochondrial diseased iPSCs express pluripotency glycoprotein and glycolipid epitopes. Flow cytometry analysis for cell surface pluripotency markers indicate expression for SSEA4 (a-c), TRA1-60 (d-f), TRA1-81 (g-i), in both control BJ-iPSC (a,d,g) and in diseased SBG6 (m.3243A>G) (b,e,h), SBG7 (m.14739G>A) (c,f,i) -hiPSCs. [file 13023_2026_4201_MOESM2_ESM.tif]

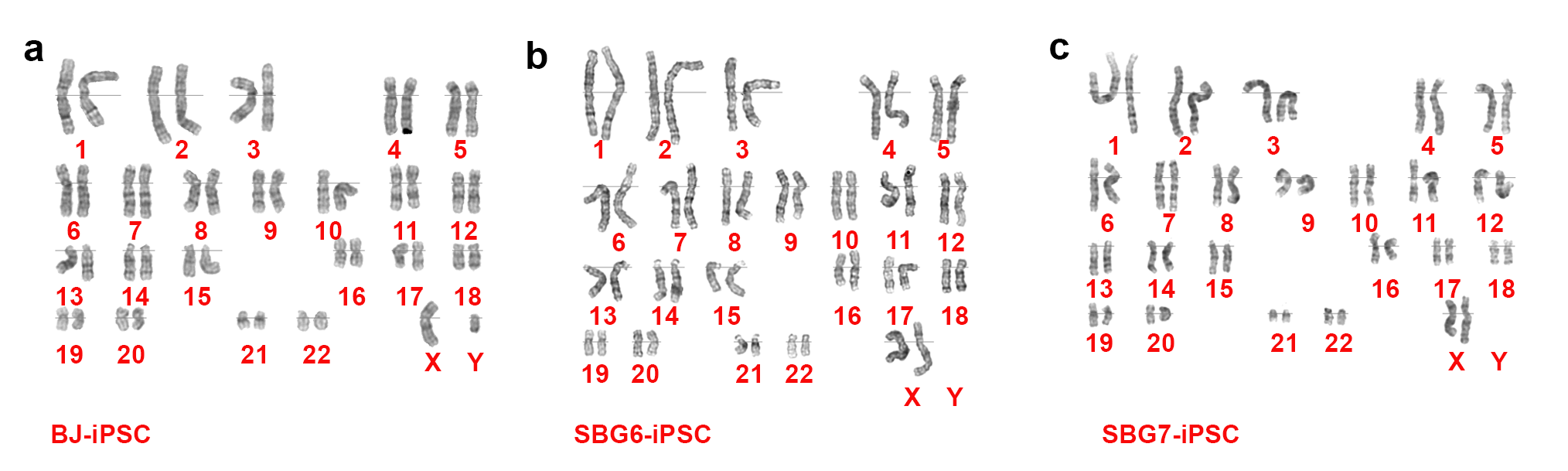

Supplement: Supplementary file 3 — Supplementary Material 3: Figure 3. Karyotype analysis demonstrated no aneuploidies or significant DNA structural abnormalities. Normal karyotype exhibited by (a) CTL-BJ-hiPSC (46, XY); (b) (SBG6-(m.3243A>G) hiPSC- 46, XX); (c) (SBG7-(m.14739G>A) hiPSC- 46, XX). [file 13023_2026_4201_MOESM3_ESM.tif]

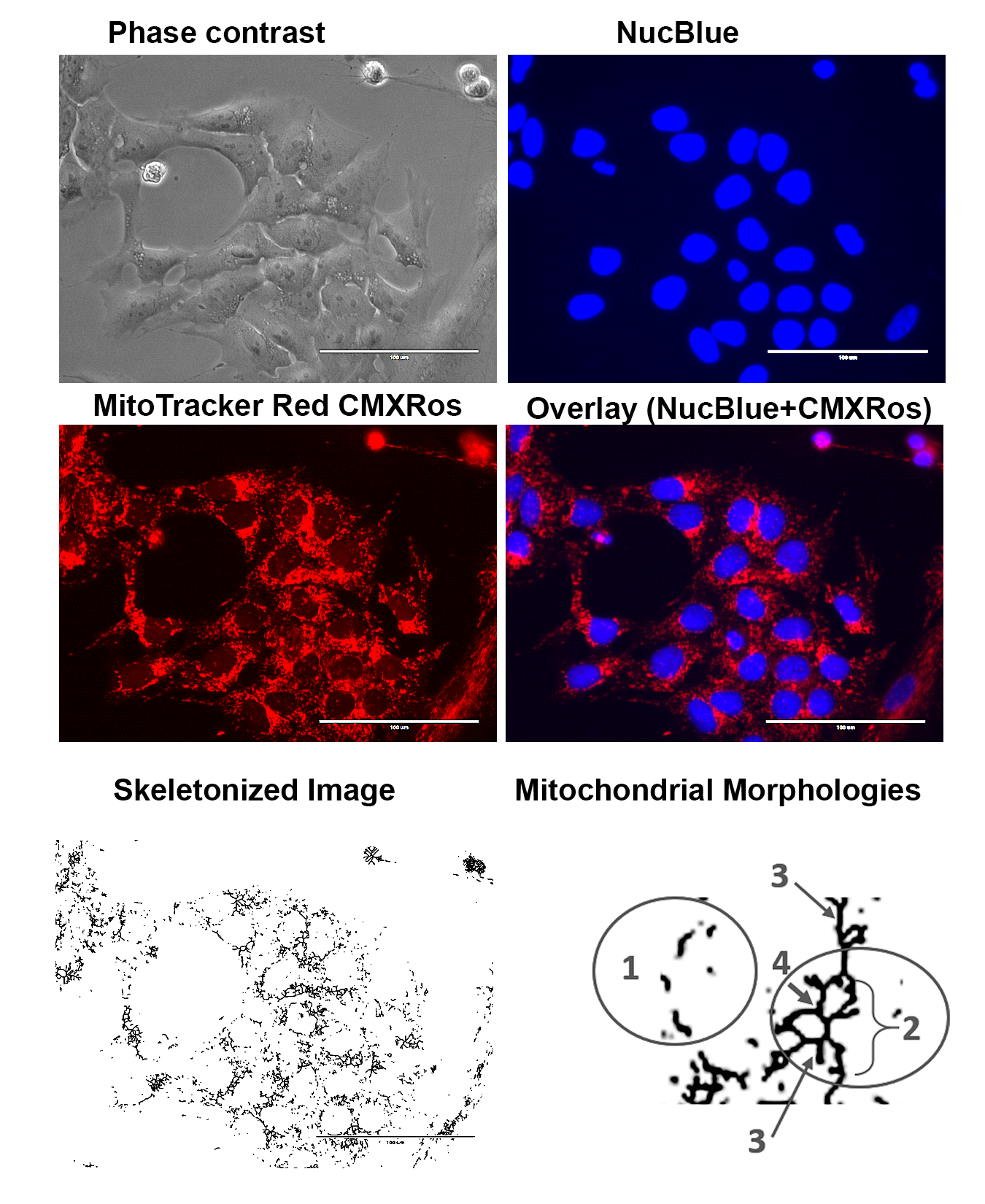

Supplement: Supplementary file 4 — Supplementary Material 4: Figure 4. Mitochondrial morphology descriptors. Representative images of control BJ-hiPSC lines stained with MTR. Phase contrast, NucBlue (nucleus), MitoTracker Red, overlay and skeletonized images control BJ-hiPSC. The mitochondrial morphology is classified as (1) individuals (1) or (2) networks. Other specific morphological structures include (3) Branch length (4) junction, which contains branches and networks. Scale bar = 100 μm. [file 13023_2026_4201_MOESM4_ESM.tif]
