# Supplementary material for: Impaired mitochondrial morphology and respiratory dysfunction in human induced pluripotent stem cells with mitochondrial tRNA mutations (m.3243A>G and m.14739G>A)
Source: Orphanet J Rare Dis. 2026 Jan 29;21:73. doi: 10.1186/s13023-026-04201-z (PMC12924315; doi:10.1186/s13023-026-04201-z)
Supplement: Supplementary file 5 — Supplementary Material 5 [file 13023_2026_4201_MOESM5_ESM.docx]

**Supplementary Table 1: Primers for Sanger sequencing**.

| **Primer Name** | **5’- 3’ Sequence** | **Product Size (bp)** |
| --- | --- | --- |
| MT-SBG6-F | CCCTGTACGAAAGGACAAGAG | 225 |
| MT-SBG6-R | TGGGTACAATGAGGAGTAGGA |  |
| MT-SBG7-F | ACCCCACAAACCCCATTACT | 219 |
| MT-SBG7-R | ATCATGCGGAGATGTTGGAT |  |
